# *PDIA2* variant associated with vitiligo

**DOI:** 10.1002/ski2.278

**Published:** 2023-08-16

**Authors:** Fucheng Li, Can Liao, Ru Li, Yongling Zhang, Xiangyi Jing, Dongzhi Li, Weiping Deng

**Affiliations:** ^1^ Department of Prenatal Diagnostic Center Guangzhou Women and Children's Medical Center Guangzhou Medical University Guangzhou China; ^2^ Department of Dermatology Guangdong Provincial People's Hospital (Guangdong Academy of Medical Sciences) Southern Medical University Guangzhou China

## Abstract

The manuscript addresses an important topic: genetic analysis of Vitiligo. Vitiligo is a complicated condition and the genetic factors account for 80% of the risk. Linkage analysis for a four generations Chinese family identified 16p13.3p13.2 as the susceptibility locus of vitiligo, whole exome sequencing then identified PDIA2 as the new candidate gene. The association between the candidate gene and vitiligo requires further investigation.

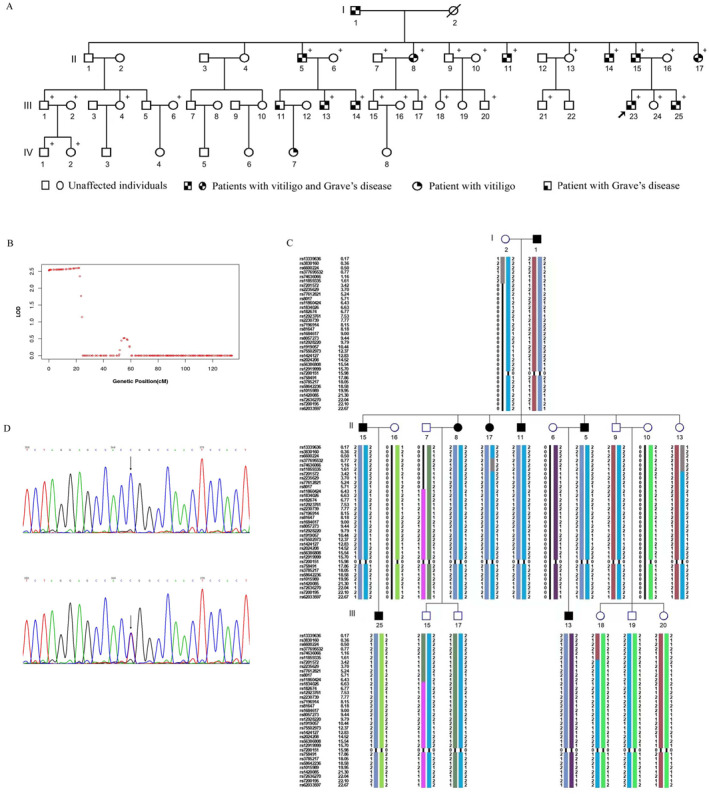

Dear Editor,

Vitiligo is a complex condition caused by melanocyte loss, leading to patches of skin or hair depigmentation in affected patients. In some patients it is accompanied by other autoimmune diseases such as thyroid disease, rheumatoid arthritis and Addison's disease.^1^ Grave's disease is one of the most prevalent thyroid diseases that coexist with vitiligo and is primarily characterised by enlarged and overactive thyroid glands.^2^ Environmental and genetic factors account for 20% and 80% of risk for vitiligo, respectively, the proportion of risk attributed to underlying genetics is greater than that of most other complex traits.^1^ Of the total genetic risk, 70 percent is attributable to common variants (minor allele frequency (MAF) > 0.01), and 30 percent is attributable to rare variants (MAF<0.01) whose identification requires family studies or genome‐wide association studies (GWAS) with extremely large sample sizes.^1^ Approximately 91% of vitiligo cases are sporadic, while ∼8% of cases have at least one affected relative in a pattern of complex inheritance.^1^ Multiple techniques have been employed to identify genes associated with vitiligo risk. GWAS is the most common approach to study the association between common variants and vitiligo susceptibility in large cohorts of sporadic cases versus unrelated controls, and more than 50 discrete loci have been identified using this analysis.^1^ Genome‐wide linkage analysis is a technique used to identify rare variants, although this requires a large family with sufficient numbers of cases.^1^ To our knowledge, only one family with confirmed cases spanning multiple generations have been reported, and chromosome 1p31.3p32.2 was identified as the vitiligo susceptibility locus in this family using linkage analysis,^3^ and subsequently *FOXD3* was identified as the candidate gene.^4^ There is currently no effective treatment for vitiligo, and the accompanying autoimmune disease may exacerbate the condition. Affected individuals often develop emotional stress. Therefore, the identification of genes associated with vitiligo is crucial for its prevention and development of personalised therapy.

In the present study, we enroled individuals from a four‐generation Chinese family with 13 affected (11 with both vitiligo and Grave's disease, one with only vitiligo and one with only Grave's disease) and 39 unaffected members (Figure 1A). The diseases were clearly inherited in an autosomal dominant pattern in this family (Figure 1A). A detailed description of the patients is summarised in Table 1. All participants were of Han Chinese ethnicity and gave written informed consent to participate in the study. The study was approved by the Ethics Committee of Guangzhou Women and Children's Medical Centre, and the principles of the Declaration of Helsinki were followed.

**FIGURE 1:**
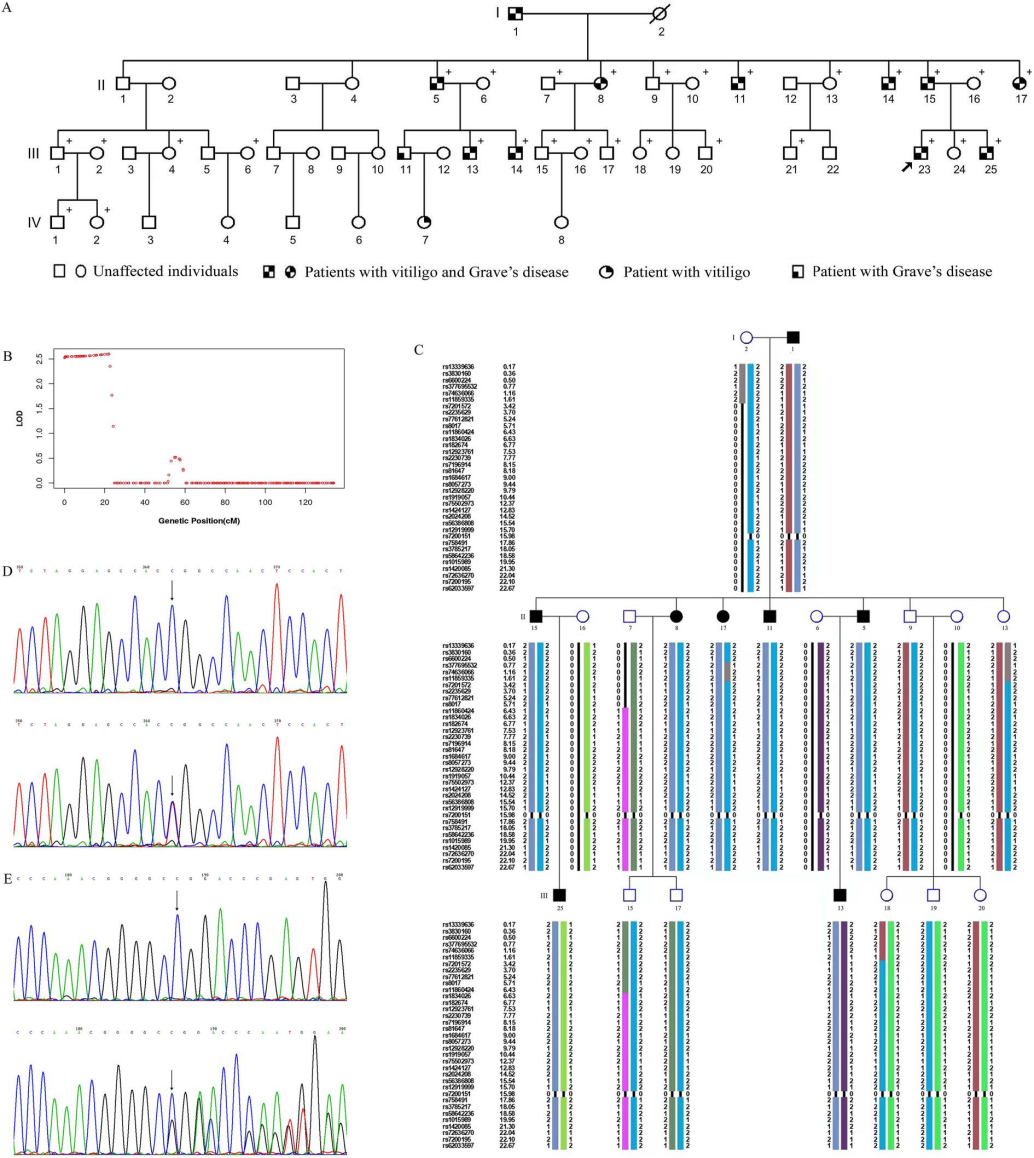
Pedigree of family with vitiligo and Grave's disease and the results of linkage analysis and whole exome sequencing. (A) The family consists of four generations with 52 members. + indicates the members available for genetic analysis. (B) Linkage analysis of the family revealed the most significant linkage (logarithm of the odds score = 2.6) in the region of 20 cM on chromosome 16. (C) Haplotype reconstruction for the family with segregation of the disease allele (shown in dark blue) in all affected family members but absent in all unaffected family members. (D) Partial sequences showing the *PDIA2* c1541C>T (p.Pro514Leu) variant. Top, forward sequences of wild‐type; bottom, forward sequence of c1541C>T (p.Pro514Leu). Arrows point to the wild‐type sequence (up) and the mutated sequence (down).

**TABLE 1:** Description of the patients with vitiligo and Grave's disease.

Patient	Gender	Age (year)	Phenotype	Age onset (year)	
				Vitiligo	Grave's disease
I:1	Male	?	Vitiligo + Grave's disease	?	?
II: 5	Male	58	Vitiligo + Grave's disease	13	22
II: 8	Female	55	Vitiligo + Grave's disease	7	18
II: 11	Male	52	Vitiligo + Grave's disease	5	20
II: 14	Male	48	Vitiligo + Grave's disease	9	17
II: 15	Male	47	Vitiligo + Grave's disease	11	19
II: 17	Female	39	Vitiligo + Grave's disease	8	19
III: 11	Male	29	Grave's disease	‐	17
III: 13	Male	23	Vitiligo + Grave's disease	11	16
III: 14	Male	19	Vitiligo + Grave's disease	9	18
III: 23	Male	20	Vitiligo + Grave's disease	14	19
III: 25	Male	11	Vitiligo + Grave's disease	7	8
IV: 7	Female	6	Vitiligo	4	‐

Genomic DNA was extracted from peripheral blood samples of 29 individuals comprising 10 affected and 19 unaffected family members using standard protocols (Qiagen, Germany). Chromosomal microarray analysis of affected family members III‐23 and unaffected II‐9 was performed. Neither gains nor losses of whole chromosomes were identified (data not shown). The Affymetrix Axiom® 2.0 Reagent Kit (Affymetrix, Inc., Santa Clara, USA) was then used to genotype 13 individuals (II‐7, II‐9, II‐11, II‐13, II‐15, II‐17, III‐13, III‐15, III‐17, III‐18, III‐19, III‐20, III‐25). A total of 6283 markers spanning the entire human genome were generated after excluding the following polymorphisms (SNPs): (1) the multiallelic probes that are not suitable for the analysis in our project, (2) the SNPs with low quality, (3) the locus with a successful genotyping rate less than 99%. Based on these single SNPs, logarithm of the odds (LOD) scores were calculated from the Kong and Cox linear model using merlin 1.1.2.^5^ The results showed a single notable region of 16p13.3p13.2 with a LOD_MAX_ of 2.6, which likely corresponded to the disease locus (Figure 1B). Haplotypes were then constructed using HaploPainter,^6^ and the locus 16p13.3p13.2 flanked by markers rs13339636 and rs62033597 (chr16: 298588–9476332) was determined to co‐segregate with the disease phenotype (Figure 1C).

To identify the causal mutation(s), we performed whole exome sequencing in six individuals, including three affected (II‐8, II‐15, III‐23) and three unaffected members (II‐9, II‐16, III‐24). The following variants were excluded: those shared by the affected and unaffected family members, those with MAF greater than 0.01 and those outside the coding region. This analysis uncovered 24 variants in 24 genes across the exome. Three of the 24 variants were located in the linked and co‐segregated haplotype, namely c.1541C>T (p.Pro514Leu) in protein disulfide isomerase family A member 2 (*PDIA2*, gene ID: 64714), c.708C>T (p.Pro236=) in hydroxyacylglutathione hydrolase (*HAGH*, gene ID: 3029) and c.711C>T (p.Ser237=) in CREB binding protein (*CREBBP*, gene ID: 1387). Sanger sequencing was performed in all family members available for genetic analysis, and the results indicated that only the *PDIA2* missense mutation c.1541C>T (p.Pro514Leu) was shared by all of the affected individuals, but absent in the remaining unaffected family members available (Figure 1D). The variant was classified to be of uncertain significance according to the American College of Medical Genetics (ACMG) guidelines.^7^

No studies on the aforementioned genes in vitiligo and Grave's disease have previously been reported. Few studies regarding *PDIA2* have been reported to date, and the biological association between this gene and disease is likewise unclear. Protein disulfide isomerases encoded by *PDIA2* are located in the endoplasmic reticulum and catalyze protein folding and thiol‐disulfide redox reactions.^8^ As endoplasmic reticulum protein, *PDIA2* was expected to have widespread roles. However, we were unable to identify any more noteworthy findings in the individuals with the variant of *PDIA2* according to the available relevant medical records, and unfortunately, the follow‐ups of the patients were lost as they were referred to us from local hospitals.

In conclusion, using linkage analysis we have identified 16p13.3p13.2 as a susceptibility region of vitiligo combined with Grave's disease. Within this loci, *PDIA2* was subsequently identified by whole exome sequencing to be candidate gene potentially involved in disease pathogenesis. Validation of this genetic association and its mechanistic involvement in vitiligo/Grave's disease pathogenesis needs further investigation.

## AUTHOR CONTRIBUTIONS

**Fucheng Li**: Conceptualisation (equal); Data curation (equal); Writing – original draft (equal). **Can Liao**: Conceptualisation (equal); Writing – original draft (equal). **Ru Li**: Data curation (equal). **Yongling Zhang**: Investigation (equal). **Xiangyi Jing**: Investigation (equal). **Dongzhi Li**: Data curation (equal). **Weiping Deng**: Conceptualisation (equal); Supervision (equal); Writing – review & editing (equal).

## CONFLICT OF INTEREST STATEMENT

No conflict of interest is declared.

## ETHICS STATEMENT

The study was approved by the Ethics Committee of Guangzhou Women and Children's Medical Centre (IRB number: 25700).

## FUNDING INFORMATION

The project is supported by Guangzhou Science and Technology Project (Grant No. 202102020061).

## Data Availability

The data that support the findings of this study are available from the corresponding author upon reasonable request.
